# Management of Refractory Metastatic Anal Squamous Cell Carcinoma Following Disease Progression on Traditional Chemoradiation Therapy

**DOI:** 10.6004/jadpro.2012.3.3.4

**Published:** 2012-05-01

**Authors:** Ninoska N. Silva, Cathy Eng

**Affiliations:** From The University of Texas MD Anderson Cancer Center, Houston, Texas

## Abstract

Case Study

Ms. S.G., a 56-year-old woman with a poorly differentiated squamous cell carcinoma of the anal canal, American Joint Committee on Cancer stage III (T2, N1, M0), was initially diagnosed in December, 2007 at an outside institution after she had noted blood in her stool for approximately 6 months. Her medical history was unremarkable. She had no known history of HIV or other sexually transmitted diseases. At the time of presentation, Ms. S.G. had an Eastern Cooperative Oncology Group performance status of 1 related to cancer-related pain. Her appetite and weight were both stable.

A complete colonoscopy demonstrated a large, immobile, ulcerated, firm, 4-cm lesion in the distal rectum, arising from the anal canal. Initial staging positron emission tomography/computed tomography (PET/CT) scan revealed a hypermetabolic inferior anorectal mass with left perirectal and presacral nodal metastases. There was no definite evidence of distant metastatic disease.

Ms. S.G. received chemoradiation treatment following her diagnostic studies, with a total dose of 45 Gy over 26 fractions to the pelvis with concurrent infusional fluorouracil (5-FU; 2, 450 mg over 7 days) and mitomycin C (12 mg/m^2^ on day 1) at an outside institution. However, during her chemoradiation therapy, Ms. S.G. experienced a 3-week treatment break due to severe radiation dermatitis, as recommended by her outside treating oncologist.

Upon treatment completion, Ms. S.G. underwent a biopsy of the anal canal, which revealed no evidence of residual malignancy. As recommended by her treating oncologist, she received four additional cycles of adjuvant infusional 5-FU in combination with leucovorin. Shortly thereafter, Ms. S.G. developed progressive pelvic pain. She underwent a second PET/CT scan, revealing mixed findings: interval resolution of abnormal standardized uptake value (SUV) activity at the primary tumor in the anal canal, but an increase in the size and SUV of nodal disease within the left perirectal and presacral regions. A CT-guided biopsy noted a perirectal abscess requiring drainage but was inconclusive for disease recurrence; Ms. S.G. was treated with IV antibiotics.

Six weeks later, repeat radiographic imaging noted additional changes suspicious for regional recurrence, which was biopsy-confirmed. Ms. S.G. was subsequently referred to MD Anderson Cancer Center for consideration of salvage pelvic exenteration.

On physical exam a mass was palpated in the left lower quadrant, but there was no evidence of inguinal adenopathy. On digital rectal exam there was notable external erythema with a fixed mass and moderate sphincter tone. A chest CT scan showed no definite evidence of metastatic disease, but an MRI of the abdomen/pelvis indicated the presence of a complex partially necrotic mass (7.6 × 4.9 × 7.3 cm^3^) extending to the rectosigmoid junction, inseparable from the left lateral bowel wall, with partial encasement of the bowel. In addition, there was infiltration of the left piriformis muscle and cervix consistent with local recurrence. She was referred to medical oncology and radiation oncology for consideration of reirradiation with concurrent neoadjuvant chemotherapy for palliation and possible surgical resection.

In early December 2008, Ms. S.G. received intensity-modulated radiation therapy (IMRT), with a total dose of 27 Gy over 18 fractions. She received concurrent infusional 5-FU at 300 mg/m^2^/day, from Monday to Friday, on the days of radiation. She also received a weekly bolus dose of cisplatin at 20 mg/m^2^. The intent was to treat to 30 Gy, but the patient deferred further treatment early due to anorectal irritation. She then underwent restaging with a PET/CT scan and a pelvic MRI in February 2009, revealing radiographic partial response of the known pelvic recurrence and reduced pelvic pain (Figures 1A and 1B).

**Figure 1:**
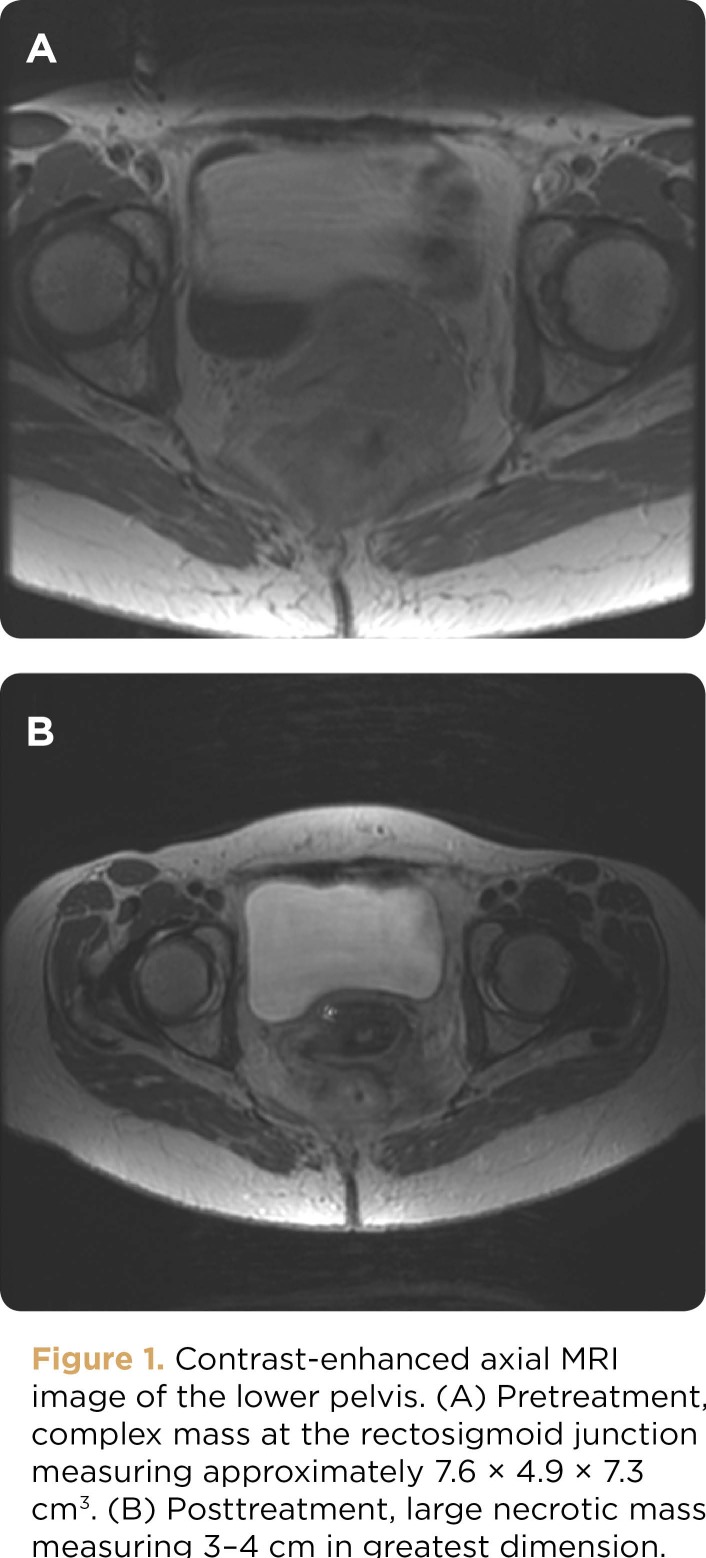
Figure 1. Contrast-enhanced axial MRI image of the lower pelvis. (A) Pretreatment, complex mass at the rectosigmoid junction measuring approximately 7.6 × 4.9 × 7.3 cm3. (B) Posttreatment, large necrotic mass measuring 3–4 cm in greatest dimension.

Figure 1. Contrast-enhanced axial MRI image of the lower pelvis. (A) Pretreatment, complex mass at the rectosigmoid junction measuring approximately 7.6 × 4.9 × 7.3 cm3. (B) Posttreatment, large necrotic mass measuring 3–4 cm in greatest dimension.

Unfortunately, in the interim, she developed multiple bilateral liver lesions and punctate pulmonary nodules consistent with distant disease (Figures 2A, 2B, and 3A).

**Figure 2:**
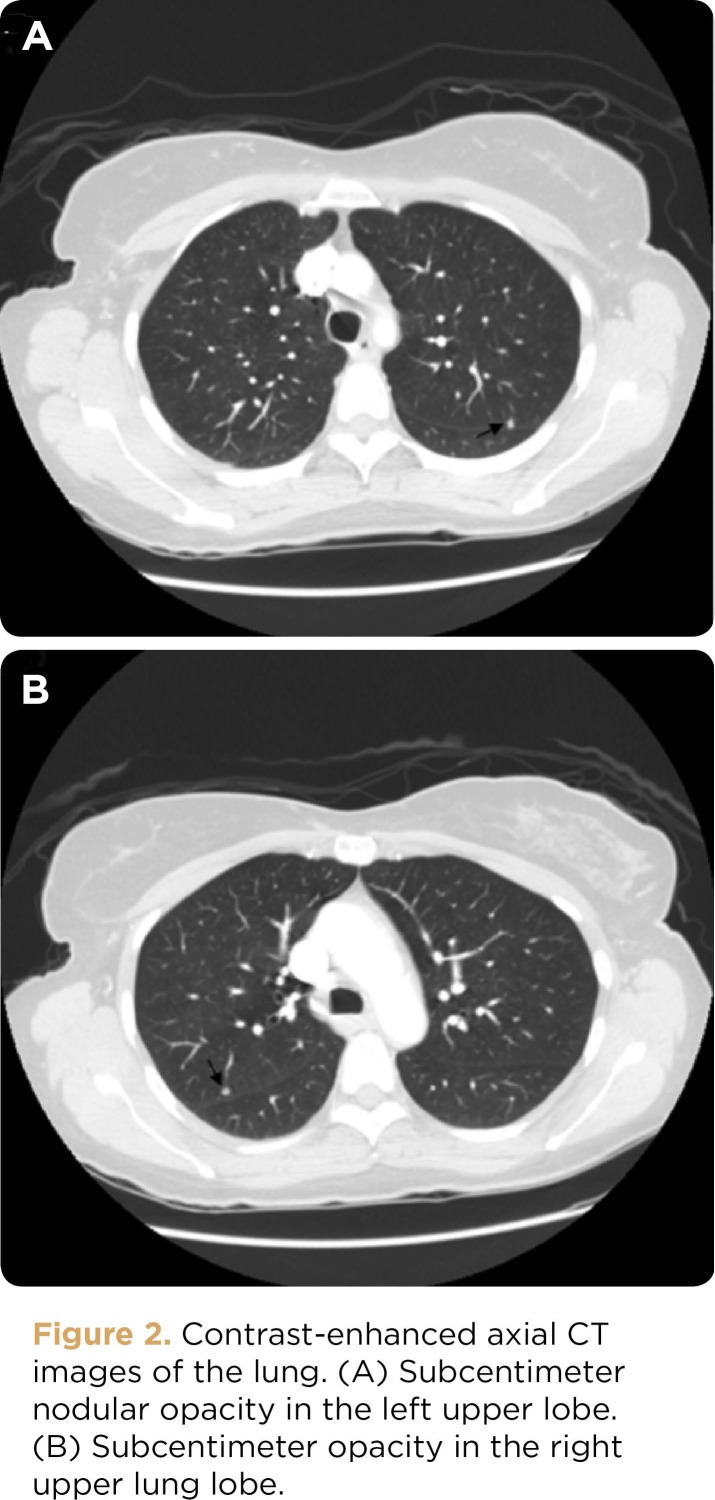
Figure 2. Contrast-enhanced axial CT images of the lung. (A) Subcentimeter nodular opacity in the left upper lobe. (B) Subcentimeter opacity in the right upper lung lobe.

**Figure 3:**
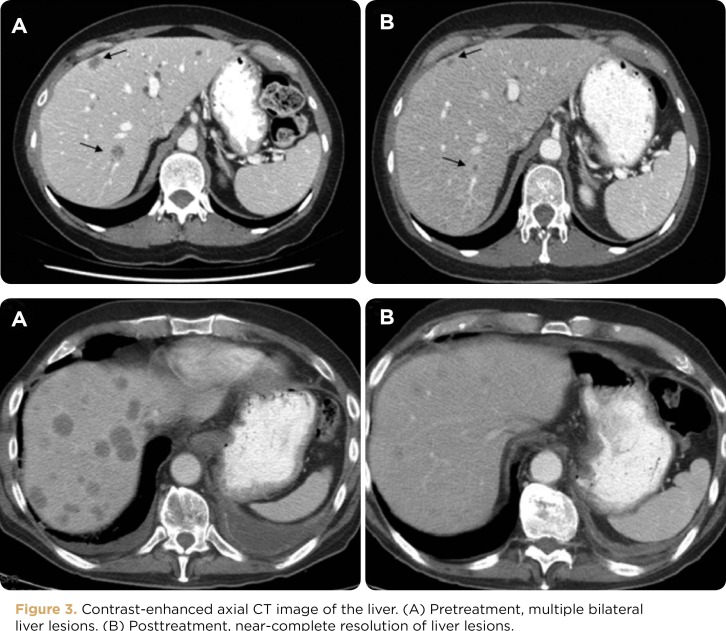
Figure 3. Contrast-enhanced axial CT image of the liver. (A) Pretreatment, multiple bilateral liver lesions. (B) Posttreatment, near-complete resolution of liver lesions.

Figure 2. Contrast-enhanced axial CT images of the lung. (A) Subcentimeter nodular opacity in the left upper lobe. (B) Subcentimeter opacity in the right upper lung lobe.

Figure 3. Contrast-enhanced axial CT image of the liver. (A) Pretreatment, multiple bilateral liver lesions. (B) Posttreatment, near-complete resolution of liver lesions.

Ms. S.G. proceeded to undergo systemic chemotherapy with carboplatin at an area under the concentration-time curve of 5 and paclitaxel at 175 mg/m^2^ day 1, every 21 days. She tolerated the treatment well. After three cycles of chemotherapy, radiographic imaging indicated a mixed response to treatment: interval resolution of the pulmonary nodules, stability of disease in the pelvic mass, but progression of the hepatic metastases.

Given Ms. S.G.'s continuing excellent performance status, further treatment was recommended. Based on recent published literature, a regimen of cisplatin at 80 mg/m^2^ day 1, vinorelbine at 25 mg/m^2^ day 1 (repeated every 28 days), and weekly cetuximab (VCC) at 250 mg/m^2^ was initiated. Remarkably, following three cycles of treatment, despite receiving multiple prior lines of chemotherapy, her restaging CT scan demonstrated complete radiographic response of the intrathoracic disease, stable response of the anorectal mass, and near-complete resolution of the hepatic lesions (Figures 3A and 3B).

Overall, Ms. S.G. had tolerated her treatment very well. Given her response and tolerability, she was evaluated again for curative surgical resection. However, she opted to receive the VCC regimen closer to home and was lost to follow-up. Unfortunately, we were unable to obtain medical records confirming if she indeed received additional treatment as recommended. Ms. S.G. was noted to have passed away due to progression of her disease approximately 6 months later.

Cancer of the anal canal is an uncommon malignancy comprising 2% of all gastrointestinal malignancies; most of these cancers are of the squamous cell histologic subtype (Jemal et al., 2008; Bilimoria et al., 2009; Horner et al., 2009). In most cases, patients present with localized disease that is often curable with concurrent chemoradiation therapy, reserving abdominal perineal resection for salvage therapy. Unfortunately, a minority of patients (12%) will have metastatic disease at initial presentation (Ryan & Willet, 2001; Horner et al., 2009). Due to the lack of evidence-based data and an unknown optimal duration of therapy, treatment decisions are often founded on more common squamous cell carcinomas (SCCs) such as cervical and lung cancer.

## Limited Data to Guide Treatment

In this article, we present the case of a patient with metastatic squamous cell carcinoma of the anal canal that progressed shortly following chemoradiation therapy with curative intent. A key point in this patient’s treatment history was the 3-week treatment delay during her concurrent chemoradiation therapy. It is well known that treatment delays should be minimized as much as possible during radiation therapy of the anal canal, and that significant delays negatively impact patient outcome (Graf et al., 2003). In addition, recent data from the phase III ACT II trial have shown no additional benefit in disease-free or overall survival for adjuvant fluorouracil (5-FU)-based chemotherapy following definitive chemoradiation therapy for curative intent (James et al., 2009).

Because metastasis from squamous cell carcinoma of the anal canal is infrequent, there are limited data to guide treatment recommendations (Table 1). The most frequently reported systemic chemotherapy regimen is 5-FU/cisplatin, which has successfully shown a partial response rate as high as 50% and a complete response rate of 15% when used to treat locally recurrent or metastatic disease (Deniaud-Alexandre et al., 2003; Ajani et al., 2008; Eng & Pathak, 2008; Jemal et al., 2008; James et al., 2009; Lynch et al., 2010). In our patient’s case, 5-FU/cisplatin was provided as an alternative due to her platinum-naive status. A well-known radiation sensitizer, 5-FU/cisplatin was provided for palliation of pain due to the primary tumor with the interim development of distant metastatic disease.

**Table 1:**
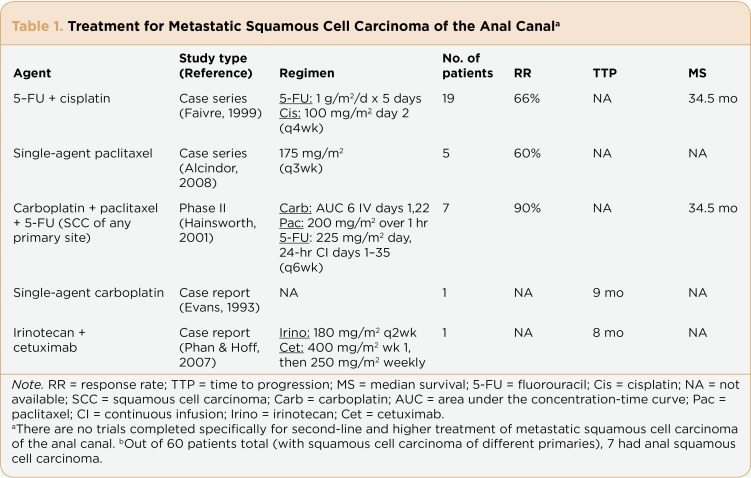
Table 1. Treatment for Metastatic Squamous Cell Carcinoma of the Anal Canal

## Trials in Cancers of Similar Histologic Subtype

Other chemotherapy regimens considered for our patient were largely based on data extrapolated from other cancers of similar histologic subtype, i.e., non–small cell lung cancer (NSCLC) and head and neck cancer (Table 2). Unfortunately, the combination of carboplatin/paclitaxel resulted in our patient’s continued distant disease progression. Therefore, we chose to pursue the therapeutic recommendation of the vinorelbine, cisplatin, and cetuximab (VCC) regimen based on data from a randomized phase II study of cisplatin and vinorelbine with or without weekly cetuximab as first-line therapy for NSCLC (Rosell et al., 2008). The vinorelbine/cisplatin combination is an accepted regimen for NSCLC, and in this phase II study the addition of cetuximab increased the response rate (35% vs. 28%), the median progression-free survival (5.0 vs. 4.6 months, HR = 0.71), and the median overall survival (8.3 vs. 7.3 months, hazard ratio = 0.71) (Rosell et al., 2008; Van Damme et al., 2008). The pivotal phase III FLEX trial (vinorelbine/cisplatin with or without cetuximab) validated the phase II findings in patients with treatment-naive stage IIIB/IV NSCLC. Those who received the cetuximab combination were found to have superior overall survival (11.3 vs. 10.1 months, *p* = .044) and response (36% vs. 29%, *p* = .010) (Pirker et al., 2009).

**Table 2:**
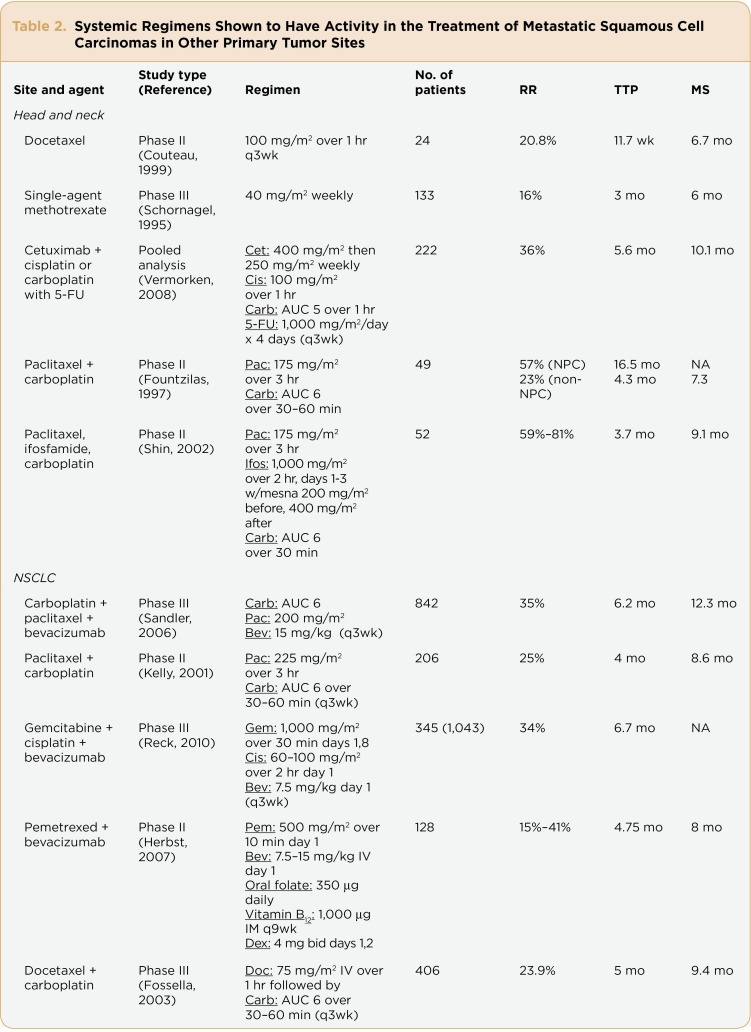
J Adv Pract Oncol 166 AdvancedPractitioner.com Table 2. Systemic Regimens Shown to Have Activity in the Treatment of Metastatic Squamous Cell Carcinomas in Other Primary Tumor Sites

**Table 2:**
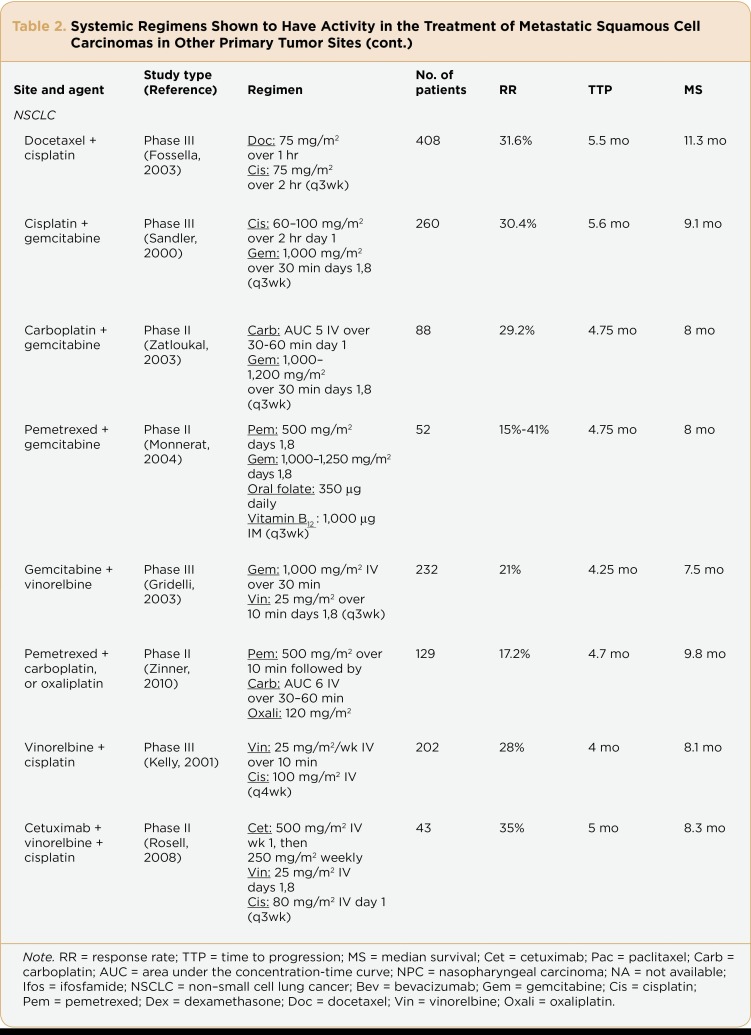
Table 2. Systemic Regimens Shown to Have Activity in the Treatment of Metastatic Squamous Cell Carcinomas in Other Primary Tumor Sites (cont.)

A second phase III trial (BMS099) evaluated the role of epidermal growth factor receptor (EGFR) inhibition in treatment-naive patients to carboplatin (AUC = 6) and either paclitaxel (225 mg/m^2^) or docetaxel (75 mg/m^2^) with a randomization to weekly cetuximab (Lynch et al., 2010). The investigators failed to fulfill the primary endpoint of progression-free survival (*p* = .236) but were able to determine improved response rate (26% vs. 17%, *p* = .007) for the investigational arm. Based on the premise of these studies, EGFR inhibition in combination with chemotherapy seems promising. Additional studies should be pursued to determine predictive markers of response. Unlike in colorectal cancer, the presence of the *KRAS* mutation does not appear to exist in SCC of the anal canal (Van Damme et al., 2008). However, extensive molecular marker analysis is limited at this time (Alvarez et al., 2006; Ajani et al., 2008).

## Conclusions

We believe this to be the first case study reported using the VCC combination in metastatic SCC of the anal canal. We recognize that a small series of case studies have been reported using cetuximab as a single agent or in combination with irinotecan as approved for use in metastatic colorectal adenocarcinoma with mild therapeutic benefit (Ajani et al., 2010). In this heavily pretreated patient, the short course of the VCC chemotherapy regimen resulted in a significant response to therapy and was well tolerated. Our treatment indicates the potential benefits of vinorelbine/cisplatin when combined with EGFR inhibition in the setting of locally recurrent or metastatic squamous cell carcinoma of the anal canal. EGFR inhibition has great promise in the treatment of metastatic squamous cell carcinoma of the anal canal, and in our patient’s case, was tolerated well.

## Acknowledgments

We would like to acknowledge Dr. Aurelio Matamoros for his assistance in radiographic imaging, Dr. Robert A. Wolff for sharing his patient experience, and Jonathan Phillips for editing.
